# Differential lipid composition and regulation along the hippocampal longitudinal axis

**DOI:** 10.1038/s41398-019-0478-6

**Published:** 2019-04-26

**Authors:** André Miguel Miranda, Francisca Vaz Bravo, Robin B. Chan, Nuno Sousa, Gilbert Di Paolo, Tiago Gil Oliveira

**Affiliations:** 1 Life and Health Sciences Research Institute (ICVS), School of Medicine, University of Minho, Braga, 4710-057 Portugal; 2 ICVS/3B’s, PT Government Associate Laboratory, Braga/Guimarães, 4710-057 Portugal; 3 Department of Pathology and Cell Biology, Columbia University Medical Center, New York City, NY 10032 USA; 4 Taub Institute for Research on Alzheimer’s disease and the Aging Brain, Columbia University Medical Center, New York City, NY 10032 USA; 5 Present Address: Denali Therapeutics Inc., South San Francisco, CA 94080 USA

**Keywords:** Molecular neuroscience, Depression

## Abstract

Lipids are major constituents of the brain largely implicated in physiological and pathological processes. The hippocampus is a complex brain structure involved in learning, memory and emotional responses, and its functioning is also affected in various disorders. Despite conserved intrinsic circuitry, behavioral and anatomical studies suggest the existence of a structural and functional gradient along the hippocampal longitudinal axis. Here, we used an unbiased mass spectrometry approach to characterize the lipid composition of distinct hippocampal subregions. In addition, we evaluated the susceptibility of each area to lipid modulation by corticosterone (CORT), an important mediator of the effects of stress. We confirmed a great similarity between hippocampal subregions relatively to other brain areas. Moreover, we observed a continuous molecular gradient along the longitudinal axis of the hippocampus, with the dorsal and ventral extremities differing significantly from each other, particularly in the relative abundance of sphingolipids and phospholipids. Also, whereas chronic CORT exposure led to remodeling of triacylglycerol and phosphatidylinositol species in both hippocampal poles, our study suggests that the ventral hippocampus is more sensitive to CORT-induced changes, with regional modulation of ceramide, dihydrosphingomyelin and phosphatidic acid. Thus, our results confirm a multipartite molecular view of dorsal-ventral hippocampal axis and emphasize lipid metabolites as candidate effectors of glucocorticoid signaling, mediating regional susceptibility to neurological disorders associated with stress.

## Introduction

Lipids are a heterogeneous group of molecules that are critical for proper brain function^1^. Although recent advances in the areas of genomics and proteomics have extensively broaden our knowledge on the molecular composition of the distinct brain regions, the study of the lipidome has been lagging behind owing to technical limitations and difficulty in translating molecular profiles to cognitive function^2–4^. Increased accessibility to approaches such as mass spectrometry (MS)-based lipidomics and in vivo manipulation of lipid signaling pathways, by means of transgenic mice or viral mediated expression of lipid enzymes, currently allows a more- comprehensive overview of the role of lipids in brain function and the identification of characteristic lipid signatures associated with specific pathological conditions^5–9^.

The hippocampus is a brain structure critically involved in cognition (learning and memory) and in emotional processing. These functions are believed to be differentially regulated by subregions along the longitudinal axis of the hippocampus, from dorsal to ventral poles in rodents, or from posterior to anterior poles in humans, respectively^10^. A dominant view in the field implicates the dorsal hippocampus particularly in cognitive function (*e.g.*, spatial memory), whereas the ventral hippocampus mediates emotional responses^11–13^. Although the basic intrinsic circuitry of the hippocampus is conserved along its longitudinal axis (*e.g.*, CA1—3 and dentate gyrus), the connectivity pattern of the dorsal and ventral hippocampus is quite distinct^10^. In addition to dissimilar extrinsic circuitry, functional differentiation of hippocampal subregions may also be imposed through distinct autonomous cellular properties. For instance, dorsal and ventral hippocampus differ in intrinsic electrophysiological properties, such as paired pulse facilitation and the ability to induce long-term potentiation^14,15^. The implementation of new genomic-scale tools has redefined the anatomical boundaries of the hippocampus along its longitudinal axis^16–18^. Although the transcriptomic and proteomic landscapes of hippocampal poles have since been linked to hippocampal regional functionality, their lipid composition has not yet been topographically characterized^19^. Recently, differential dorsal-ventral sphingolipid metabolism was implicated in the mediation of hippocampus-dependent behavioral tasks, broadening the importance of correlating the biochemical and functional role of lipids in brain function^20^.

Stress can be perceived as a challenge to an individual’s homeostasis. Intense or prolonged exposure to stressors are known to elicit deleterious adaptive mechanisms, with particular functional and structural impact in the hippocampus^21,22^. Although stressful events are associated with a variety of mental disorders, chronic stress in rodents induces responses such as dendritic atrophy, depressive- and anxious-like behavior and learning and memory deficits^22,23^. A significant portion of the pathological effects of chronic stress are believed to be mediated by prolonged release of glucocorticoids (GC), namely corticosterone (CORT) in rodents. Although stress is known to induce remodeling of the hippocampal intrinsic and extrinsic circuitry, it is possible that some of the functional phenotypes may rely on a bimodal response mediated by the dorsal and ventral hippocampus, respectively. Interestingly, CORT receptors are differentially expressed along the hippocampal axis. High-affinity mineralocorticoid receptors (MR) and low-affinity GC receptors (GR) are each more highly expressed in the ventral and dorsal hippocampus, respectively^24^. The distinct affinity of receptors to CORT may thus explain a more-homeostatic role for MR in basal conditions and on-demand GR activation in response to increased levels of CORT. Differential receptor expression is hence instrumental for the deleterious impact of chronic stress and may underlie regional responsivity of dorsal and ventral hippocampus to stress stimuli^25^. Recently, we have found that a chronic unpredictable stress paradigm inversely affected dorsal and ventral hippocampus CA3 arborization (atrophy vs hypertrophy, respectively), whereas only the ventral hippocampus displayed altered synaptic plasticity, namely decreased long-term depression^14^. In light of additional evidence for differential responses to stimuli along the longitudinal hippocampal axis, it is therefore critical to identify new molecular pathways that determine hippocampal regional identity and plasticity, particularly in response to stress and CORT signaling.

To test whether lipid composition can also be used as a molecular marker of the distinct longitudinal anatomical sections of the hippocampus, we performed a broad-scale lipid analysis of dorsal, intermediate and ventral hippocampus and compared it with other brain areas. We found that the hippocampus shows a continuous gradient along its longitudinal axis, with a greater distinction between the dorsal and ventral subregions. Moreover, whereas CORT treatment significantly affected both regions, we found region-specific modulation of lipid species, suggesting distinct action of lipid enzymes between the dorsal and ventral hippocampus. Altogether, our findings support a biochemical model in which the hippocampus can be partitioned in subregions based on lipid composition.

## Materials and methods

### Animals and treatments

All animal experiments were conducted in full compliance with European Union Directive 86/609/EEC and National Institutes of Health guidelines on animal care and experimentation. Adult 2-month-old male Wistar rats (Charles River Laboratories) were housed in groups of two under standard laboratory conditions (lights on from 8:00 A.M. to 8:00 P.M.; room temperature 22 °C; *ad libitum* access to food and drink). Animals were assigned to daily handling (CTRL; control group); a 4-week protocol of daily subcutaneous injections with vehicle (VEH; sesame oil) or synthetic CORT (40 mg/kg) (Sigma-Aldrich)^26^.

### Lipids analysis

Animals were killed by decapitation, brains immediately macrodissected, frozen in liquid nitrogen and stored at − 80 °C until further processing. Lipids were extracted using a modified Bligh-Dyer procedure. In brief, frozen tissue was resuspended and homogenized in 750 µL ice-cold methanol:chloroform (2:1, v/v). Phase separation was obtained by addition of 375 µL chloroform and 250 µL 1 m potassium chloride to a final solution ratio methanol:chloroform:potassium chloride 2:2.5:1 (v/v) and centrifugation for 2 min at 13,000 rpm. The lower organic phase was concentrated in a nitrogen dryer, resuspended in 15 µL chloroform and spiked with appropriate internal standards (see below) for further analysis using a 6490 Triple Quadrupole LC/MS system (Agilent Technologies), as previously described^8,26^. Glycerophospholipids and sphingolipids were separated using an Agilent Zorbax Rx-Sil column (diameter 2.1 × 100 mm) under an initial mobile phase A (chloroform:methanol:1 m ammonium hydroxide, 89.9:10:0.1, v/v/v) followed by phase B (chloroform:methanol:water:1 m ammonium hydroxide, 55:39.9:5:0.1, v/v/v/v); 95% A for 2 min, linear gradient to 30% A over 18 min and held for 3 min, and linear gradient to 95% A over 2 min and held for 6 min. Sterols and glycerolipids were separated with reverse-phase high-performance liquid chromatography using an isocratic mobile phase chloroform:methanol:0.1 m ammonium acetate (100:100:4v/v/v) at a flow rate of 250 μl/min with an Agilent Zorbax Eclipse XDB-C18 column (diameter 4.6 × 100 mm)^8^. Individual lipid species were measured by multiple reaction monitoring transitions and lipid concentration was calculated by referencing to appropriate internal standards: D5-cholesterol, CE 17:0, 4ME 16:0 diether DG, D5-TG 16:0/18:0/16:0, SM d18:1/12:0, dhSM d18:0/12:0, Cer d18:1/17:0, GalCer d18:1/12:0, LacCer d18:1/12:0, Sulf d18:1/17:0, PA 14:0/14:0, PC 14:0/14:0, PE 14:0/14:0, PG 15:0/15:0, PS 14:0/14:0, LPC 17:0, LPE 14:0, LPI 13:0, BMP 14:0/14:0 (Avanti Polar Lipids) and PI 16:0/16:0 (Echelon Biosciences). Lipid classes not internally standardized owing to commercial unavailability of respective standards were referenced to closely eluted standards. Ether-linked species (*N*-acyl-PS and GM3) were normalized to corresponding acyl-linked standards (PS 14:0/14:0 and PI 16:0/16:0, respectively). Lipid concentration was normalized by molar concentration across all species detected for each sample, and expressed as relative mean mol%. Lipid nomenclature follows LIPID MAPS consortium guidelines and lipid species are annotated as lipid classes followed by total number of carbons and degree unsaturation of respective acyl chains (*e.g.*, PC 30:0)^27^. DG and TG species are annotated as mentioned with the addition of acyl carbon and unsaturation of the product ion (*e.g.*, DG30:0/14:0). Sphingolipids contained d18:1 long-chain base except dhSM species, containing a d18:0 base. *N-a*cyl-PS species contained C16:0 *N*-linked acyl chains. Nomenclature abbreviations are FC, free cholesterol; CE, cholesteryl ester; DG, diacylglycerol; TG, triacylglycerol; Cer, ceramide; SM, sphingomyelin; dhSM, dihydrosphingomyelin; HexCer, hexosylceramide; Sulf, sulfatides; Sulf(2OH), 2-hydroxy *N*-acyl sulfatide; LacCer, lactosylceramide; PA, phosphatidic acid; PC, phosphatidylcholine; PCe, ether phosphatidylcholine; PE, phosphatidylethanolamine; PEp, plasmalogen phosphatidylethanolamine; PG, phosphatidylglycerol; PI, phosphatidylinositol; PS, phosphatidylserine; LPC, lysophosphatidylcholine; LPCe, ether lysophosphatidylcholine; LPE, lysophosphatidylethanolamine; LPEp, plasmalogen lysophosphatidylethanolamine; LPI, lysophosphatidylinositol; BMP, bis(monoacylglycero)phosphate; *N*-acyl-PS, *N*-acyl-phosphatidylserine.

### Statistical analysis

Statistical analysis was performed using Prism 6.0 software (Graphpad Software). Animals were randomly assigned in experimental groups. No statistical method was used to determine sample size and was based on previous studies^26^. Samples were blinded during tandem biochemical analysis and unblinded following data processing. Normal distribution was tested using Kolmogorov–Smirnov testing and homogeneity of group variances confirmed. All data are given as mean ± s.e.m. for a given *N* of biological replicates (see each figure legend for exact details). A confidence interval of 95% was assumed for all statistical tests. For comparison of two experimental conditions, unpaired two-tailed Student’s *t* test was performed. Descriptive statistics and individual *p* values are listed as Supplementary Tables. No samples or animals were excluded from analysis.

## Results

### Regional lipid heterogeneity in the rodent brain

For a better understanding of the lipid composition of the rodent brain, we performed a comprehensive MS-based lipidomic analysis of distinct brain regions, namely the hippocampus, prefrontral cortex (PFC), amygdala, and cerebellum in male Wistar rats injected with VEH (see Methods for details). Based on the literature supporting molecular and functional heterogeneity along its longitudinal axis, we subdivided the hippocampus in dorsal, intermediate and ventral portions^10^.

**Fig. 1:**
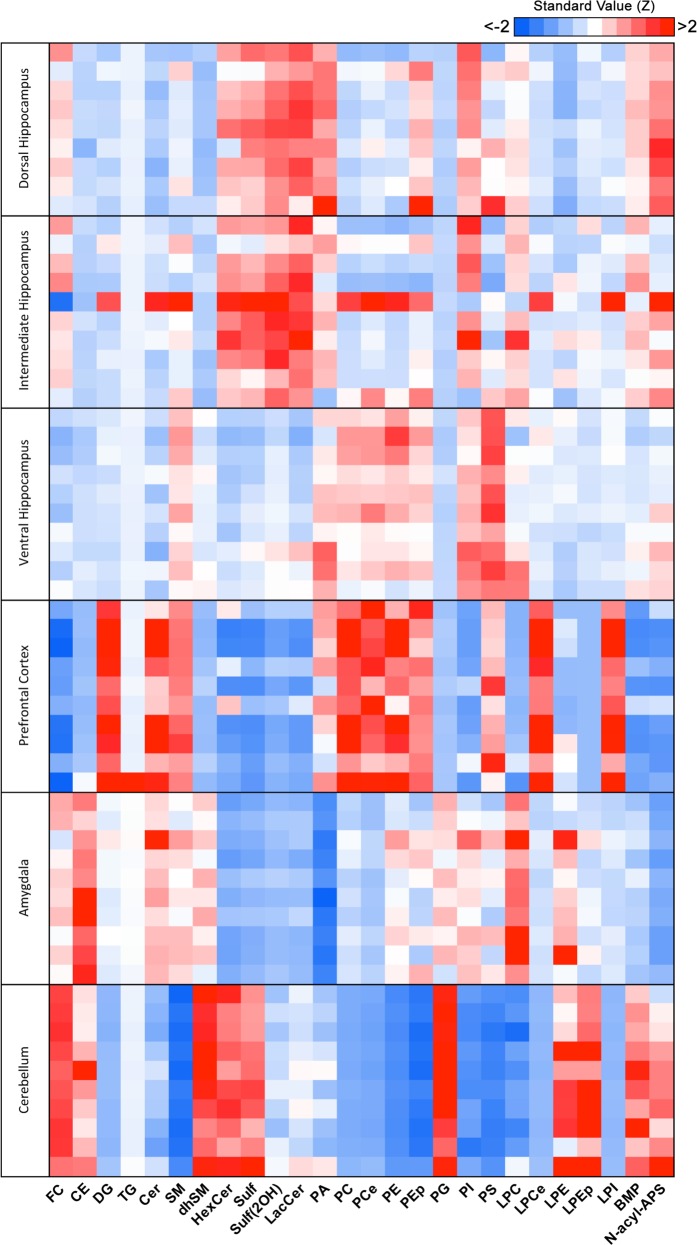
Lipidomic analysis of brain regions from adult rats. Adult rats were subjected to daily injections of vehicle (VEH) and brain regions were macrodissected prior to analysis by LC-MS (see Methods for details). Heatmap generated by calculating the standard value of each lipid class (column) using as reference the pooled average relative mol% of all brain regions. *Z* scores [(mol% of lipid class of each animal−average mol% of lipid class of all brain regions of pool of animals)/standard deviation of average mol% lipid class of all brain regions of pool of animals] represented in gradient color; blue indicates negative *Z* value; red indicates positive *Z* value (lower and higher than reference average, respectively). Each row indicates an individual animal, per brain region (*N* = 9 for dorsal hippocampus, *N* = 10 for all other regions)

In order to analyze the compositional similarity and disparity between regions, we plotted a heatmap of standard scores (*Z* score) of the average mol% of each lipid class per region, taking as reference value the mean of all regions pooled (Fig. 1). First, we observed a high degree of similarity, expressed as lower modular *Z* scores (lighter blue and red shades), between the three hippocampal regions (dorsal, intermediate, and ventral) relatively to the other three brain regions (PFC, amygdala, and cerebellum) under study. More specifically, we found the dorsal hippocampus to show higher similarity with the intermediate than the ventral hippocampus, consistent with the proposed continuous gradient of molecular identity along dorsal-ventral axis, where distinctions are more obvious between the poles^16,17^. The other three brain regions showed a high degree of differentiation comparatively to the hippocampal subregions and between themselves.

Specifically, the dorsal and intermediate hippocampus showed great enrichment in glycosphingolipids HexCer, LacCer, and Sulf. The PFC showed the highest abundance of glycerophospholipids, such as PC, PCe, PE, PEp, and lysolipid derivatives, LPCe and LPEp. The amygdala was particularly enriched in CE and LPC, whereas the cerebellum showed the highest enrichment in FC, glycosphingolipids, the atypical glycerophospholipid BMP and its precursor PG. Our findings were confirmed and validated by performing the same analysis in a previously published cohort of control adult rats (CTRL; not injected with VEH) (Supplementary Fig. 1)^26^. Altogether, our results highlight the heterogeneity of lipid composition between distinct brain regions and identify a continuous molecular gradient along the longitudinal axis of the hippocampus.

### Distinct lipid signature of dorsal and ventral hippocampus

**Fig. 2:**
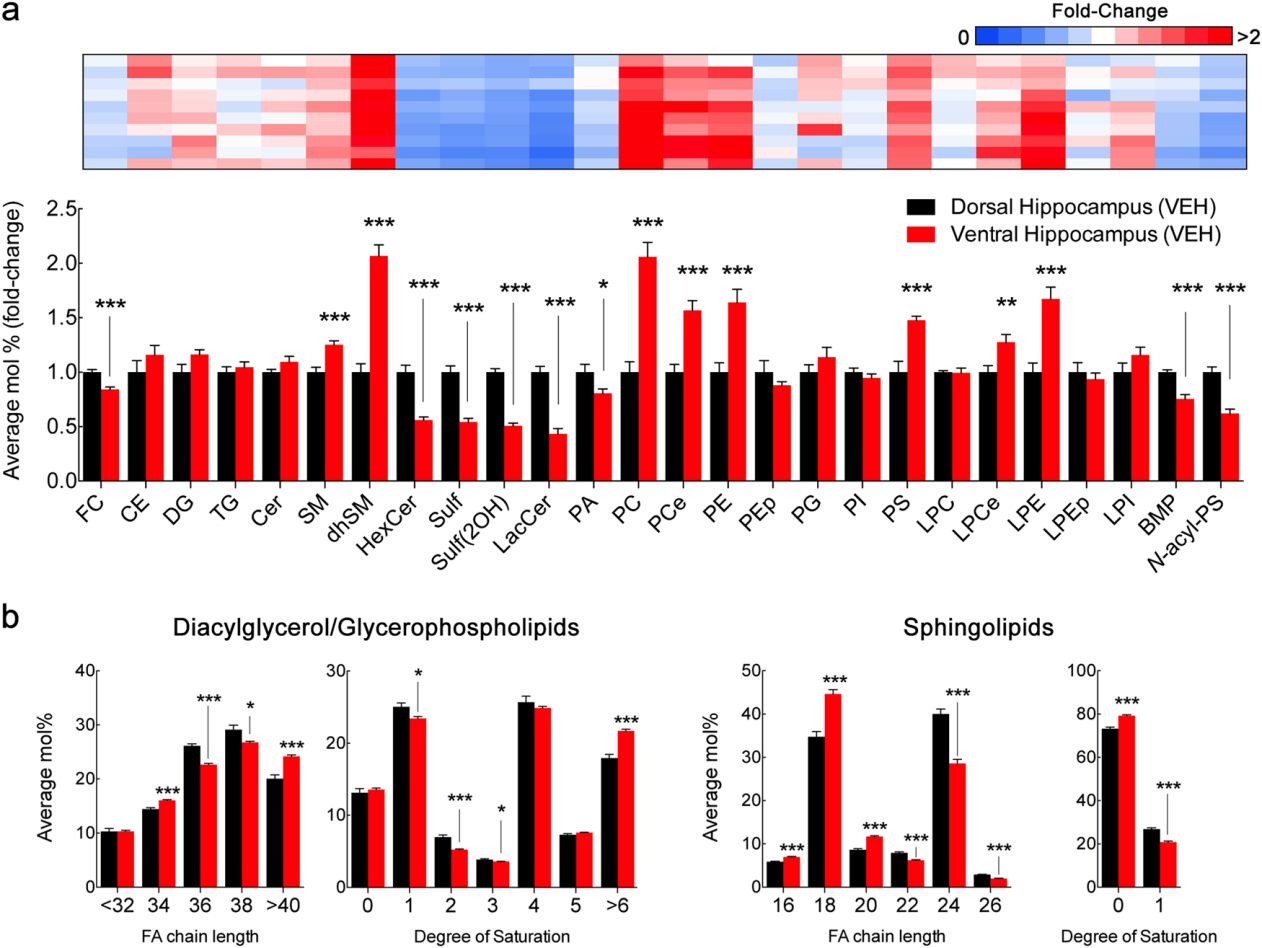
Distinct lipid composition of dorsal and ventral hippocampus in adult rats. **a** LC-MS analysis of dorsal and ventral hippocampus macrodissected from adult rats injected with vehicle (VEH). For lipid nomenclature, see Methods section. Bar graphs indicate fold-change of average relative mol% of all lipids measured, normalized to dorsal hippocampus (mean ± SEM, *N* *=* 9 for dorsal hippocampus, *N* = 10 for ventral hippocampus). Upper panel, heatmap indicates individual plot of normalized average mol% of each lipid class in the ventral hippocampus. Values represented in gradient color, blue indicates below 1 and red indicates above onefold-change, respectively, normalized to dorsal hippocampus. Descriptive statistics listed in Supplementary Table 1. *p *<* 0.05, **p *<* 0.01, and ***p < 0.001 in two-tailed Student’s *t* test. **b** LC-MS analysis of diacylglycerol/glycerophospholipid and sphingolipid acyl chain. Values expressed as average mol% of total lipid measured, normalized to dorsal hippocampus. Lipids were classified per total acyl carbons and degree of unsaturation (mean ± SEM, *N* = 9 for dorsal hippocampus, *N* = 10 for ventral hippocampus). Descriptive statistics listed in Supplementary Table 2. *p *<* 0.05, **p *<* 0.01, and ***p < 0.001 in two-tailed Student’s *t* test

We subsequently focused our analysis on directly comparing the composition of the dorsal and ventral hippocampus (Fig. 2a; Supplementary Table 1). In VEH animals, the ventral hippocampus showed reduced levels of FC, as well as sphingolipids HexCer, LacCer, and Sulf and the atypical phospholipids BMP and *N*-acyl-PS comparatively to the dorsal hippocampus. On the contrary, the ventral hippocampus was enriched in SM and dhSM, as well as in glycerophospholipids such as PC by nearly twofold and PCe, PE, PS, LPCe, and LPE by 1.5-fold. Of note, whereas PC levels were increased, PA, both precursor and product of PC hydrolysis, was significantly decreased by ~ 20%. Importantly, overlapping results were observed in CTRL rats, with the exception of lack of significant changes between hippocampal subregions in PCe, PE (*p* = 0.08 and 0.06, respectively). Possibly owing to reduced levels of PE in the ventral hippocampus in CTRL rats, higher levels of LPE were not detected and LPEp were significantly reduced by 25% (Supplementary Fig. 2; Supplementary Table 2).

Although lipid classes are generally defined and classified by their polar head group, the acyl chains linked to their backbone show great variety in length and saturation, with important functional implications^28^. In general, the degree of length and saturation provides distinct physical properties to membranes, namely thickness, fluidity, and microdomain assembly^29^. Therefore, we analyzed the acyl composition of glycerophospholipids/DG and sphingolipids in VEH animals (Fig. 2b; Supplementary Table 3). We found that the ventral hippocampus was enriched in glycerophospholipids containing both shorter 34 carbon (C34) and over C40 acyl chains, at the significant expense of intermediate C36 and C38 chains. Moreover, glycerophospholipids from this region also showed higher abundance of poly-unsaturated chains with over 6 double bonds, at the expense of less saturated lipid species, namely containing 1–3 double bonds. Inversely, sphingolipids from the ventral hippocampus were significantly shorter, with higher abundance of C16–20 acyl chains and reduced levels of C22–26, comparatively to the dorsal portion. Contrarily to glycerophospholipids, sphingolipids were more saturated in the ventral than in the dorsal hippocampus.

Owing to the bimodal distribution of metabolically related lipids classes (e.g., PC, PA, and DG) in both experimental groups (CTRL and VEH), we directly compared the abundance of all lipid species detected by LC-MS (see Supplementary Fig. 3 for all lipid species detected). In both experimental groups, we detected an inverse profile of Cer species in the ventral and dorsal hippocampus, with longer and shorter acyl chains respectively, whereas no prominent acyl chain enrichment was detected for other sphingolipids in general. The ventral hippocampus showed significantly decreased levels for most PA species identified, particularly those with shorter acyl chains (C32–38) and decreased unsaturation (0–2 double bonds), similarly to PI species (C34–26 and 1–3 double bonds). Inversely, the ventral hippocampus was enriched in PC species with no predilection for specific acyl chain profile. Therefore, the dorsal and ventral hippocampus differ significantly in their lipid composition, with decreased levels of glycosylated sphingolipids and PA specifically in the ventral area, whereas acyl chain composition of glycerophospholipids/DG and sphingolipids vary inversely, with longer/unsaturated and shorter/saturated acyl chains, respectively.

### CORT treatment affects the dorsal and ventral hippocampus differentially

Chronic stress exposure culminates in the release of GC, which mediate a myriad of pathologic effects of prolonged stress. Thus, to test whether high CORT levels modulate the lipid landscape of the hippocampus in a region-specific manner, we subjected a group of animals to daily subcutaneous injections of synthetic CORT^30,31^.

**Fig. 3:**
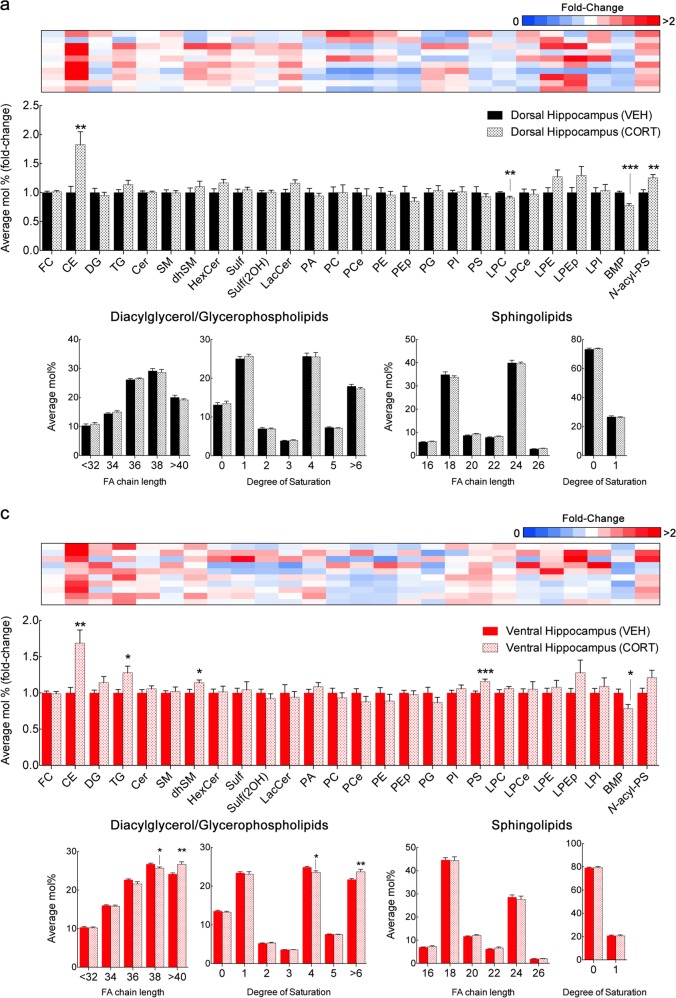
Effect of corticosterone injections in the lipid composition of dorsal and ventral hippocampus. **a**, **b** LC-MS analysis of dorsal and ventral hippocampus macrodissected from adult rats submitted to a 4-week protocol of daily injections of corticoterone (CORT) compared with age-matched control injected with vehicle (VEH). For lipid nomenclature, see Methods section. Bar graphs indicate fold-change of average relative mol% of all lipids measured, normalized to VEH (mean ± SEM, *N* *=* 9 for dorsal hippocampus of VEH; *N* = 10 for remaining groups). Upper panels, heatmap indicates individual plot of normalized average mol% of each lipid class in CORT-treated animals. Values represented in gradient color, blue indicates below 1 and red indicates above onefold-change, respectively, normalized to VEH. Descriptive statistics listed in Supplementary Tables 4 and 5. *p *<* 0.05, **p *<* 0.01, and ***p < 0.001 in two-tailed Student’s *t* test

**Fig. 4:**
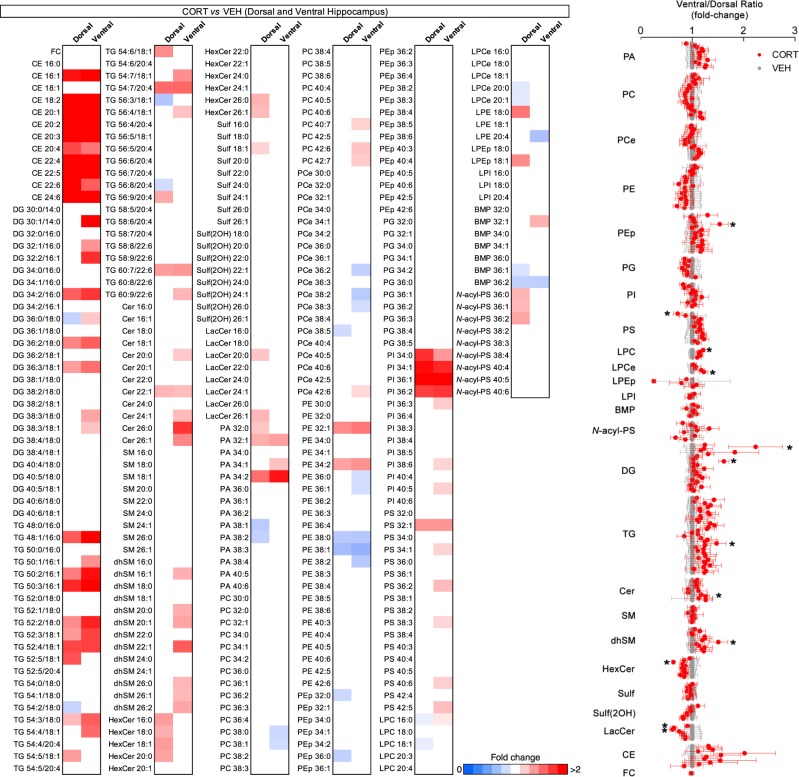
Specific lipid species are differently regulated in the dorsal and ventral hippocampus by corticosterone. **a** Lipidome profile of dorsal and ventral hippocampus from adult rats submitted to daily injections of corticosterone (CORT) compared with vehicle-treated animals (VEH). The heatmap represents all lipid species detected by LC-MS in macrodissected tissue. Lipids were classified per total acyl carbons and degree of unsaturation, respectively. For additional lipid nomenclature, see Methods section. Values represented as average fold-change of individual species in dorsal and ventral hippocampus of CORT animals, normalized to VEH (only statistically significant values shown (*p* < 0.05), *N* = 9 for dorsal hippocampus of VEH; *N* = 10 for remaining groups). Results were compared using two-tailed Student’s *t* test. **b** A ventral/dorsal hippocampus ratio for each lipid species detected, per animal, was calculated in adult rats injected with VEH (gray) or CORT (red) (mean ± SEM, *N* *=* 9 for VEH; *N* = 10 for CORT). *p *<* 0.05, **p *<* 0.01, and ***p < 0.001 in two-tailed Student’s *t* test

In the dorsal hippocampus, the most prominent change at the lipid class level was a significant near twofold increase in CE (Fig. 3a; Fig. 4a; Supplementary Table 4). Levels of LPC and BMP were decreased by 10–20% and *N*-acyl-PS increased by 25% (Fig. 3a; Supplementary Table 4). In addition, we found increases in multiple species of other classes of neutral lipids, namely DG and TG, though no specific acyl chain pattern was detected (Fig. 4a). We also detected multiple increases in HexCer species, with both shorter and longer acyl chains (C16–20 and C26) (Fig. 4a). Curiously, PA species showed a bimodal distribution with increases in shorter species, namely 32:0, 32:1, and 34:2, whereas 38:1 and 38:2 were decreased (Fig. 4a). Likewise, increases in shorter PI species (34:0, 34:1, 36:1, and 36:2) were identified (Fig. 4a).

In the ventral hippocampus, CORT also induced an increase in CE and a decrease in BMP levels, matching the phenotype seen in the dorsal region (Fig. 3b; Supplementary Table 4). Concerning neutral lipids, we detected a greater number of DG species affected, as well as increased levels of TG (Fig. 3b; Fig. 4a; Supplementary Table 4). Contrarily, although HexCer species were unchanged in the ventral hippocampus, increases were found in multiple Cer and dhSM species (Fig. 4a). Specifically, overlapping acyl chain profiles were detected in the latter lipid classes, particular Cer and dhSM 22:0, 26:0, 26:1, suggesting a subset of species from these classes to be specifically regulated by CORT signaling. Notably, only longer Cer species (C20–26) were increased in ventral hippocampus, but not shorter Cer (C16–18) (Fig. 4a). Moreover, in contrast to the dorsal region in which longer PA C38 species were decreased, in the ventral hippocampus only shorter PA species were increased, namely 32:1, 34:1, and 34:2 (Fig. 4a). Finally, the same PI species (C34–36) found to be increased in the dorsal hippocampus were also upregulated in the ventral pole after CORT treatment, in addition to multiple unsaturated PS species (32:1, 34:1, 36:2, 38:3, 40:5, 40:6, and 42:5) (Fig. 4a). Concerning the acyl chain composition of glycerophospholipids/DG and sphingolipids, no significant changes were observed in the dorsal hippocampus upon CORT exposure (Fig. 3a; Supplementary Table 5), whereas the ventral hippocampus presented increased levels of glycerophospholipids/DG longer than C40 and over six double bonds, at the expense of lipids containing four double bonds (Fig. 3b Supplementary Table 5).

Considering the region-specific impact of CORT treatment in particular lipid species (e.g., DG, TG, Cer, and HexCer), we calculated the ratio of the abundance of each of the lipid species detected in both hippocampal regions and the degree of biochemical coherence between VEH and CORT animals (Fig. 4b). We found that 12 out of ~ 300 species had their coherence significantly changed (PEp 36:0, PS 32:0, LPC 16:0, LPCe 20:1, DG30:1/14:0, DG36:0/18:0, TG 56:3/18:1, Cer 24:1, dhSM 22:1, HexCer 18:1, Lac18:0, and LacCer 20:0) (Fig. 4b). Thus, our results show that elevated levels of CORT lead to major significant changes in neutral lipids in both dorsal and ventral hippocampus, whereas specific glycerophospholipids and sphingolipids species are disturbed in a region-specific manner, with more pronounced sensitivity in the ventral hippocampus in response to CORT treatment.

## Discussion

Here, we present the first detailed analysis of the lipid composition along the hippocampal longitudinal axis. Initially, we compared the subregions of the hippocampus (dorsal, intermediate and ventral) with other brain regions, namely the PFC, amygdala and cerebellum (Fig. 1). Consistent with the preserved intrinsic architecture of the hippocampus, we found higher similarity (*i.e.*, less variance) between the different hippocampal subregions than other brain regions^10^. Importantly, we found higher similarity between the dorsal and intermediate hippocampus than its ventral component (Fig. 1). Furthermore, we found that CORT, a known mediator of chronic stress exposure, selectively impacts the lipid composition of dorsal and ventral hippocampus respectively (Figs. 3 and 4).

The comparison of the lipid composition between dorsal and ventral hippocampus in two groups of adult rats (CTRL and VEH) revealed robust and reproducible observations (Supplementary Fig. 3). Specifically, we found an enrichment for complex glycosphingolipids in the dorsal hippocampus (HexCer, Sulf and LacCer), at the expense of simpler sphingolipids (SM and dhSM). Notably, the ventral hippocampus showed increased levels of shorter Cer C16–20 and reduced levels of longer Cer C22–26. De novo sphingolipid synthesis is initiated by the action of serine palmitoyltransferase and sequential addition of an acyl chain generates Cer. Remarkably, six distinct ceramide synthases are known to catalyze the formation of multiple Cer species owing to different affinity to acyl-CoA substrates^32^. Our observations suggest that Cer biosynthesis in the ventral hippocampus likely depends on ceramide synthase isoforms 1 and 6, which preferably use 18:0 and 16:0 respectively. Alternatively, activation of hydroxilases or elongases in the dorsal hippocampus may account for increased longer and unsaturated Cer species (*e.g.*, Cer C22–26). We also found that the ventral hippocampus was enriched in glycerophospholipids (PC, PCe, PE, and PS), except for PA (Fig. 2). Considering these observations, it will now be crucial to identify the mechanisms underlying regional molecular identity. A likely candidate is selective expression of lipid-metabolizing enzymes as a result of regional transcriptomic signatures, although mRNA expression does not always correlate with either protein expression or enzyme activity, also dependent on post-translational modifications and appropriate subcellular milieu for optimal function^32,33^. Thus, to further understand the impact of regional lipid composition, an enzyme-centric approach through the use of gain- and loss-of-function genetic and pharmacological tools may tailor designed lipid compositions to later map molecular signatures to function, such as electrophysiological plasticity or behavior^20,34–36^. To complement our approach using macrodissected tissue, future studies should implement new techniques such as matrix-assisted laser desorption/ionization imaging mass spectrometry to profile brain regions in situ, the hippocampus in particular, with cellular resolution^37,38^. Likewise, single-cell tissue dissociation and analysis will be crucial to resolve the individual contribution of neurons and glial cells to regional lipid distribution, as recently performed in the field of transcriptomics^39,40^.

Next, we studied the impact of elevated CORT levels, a critical mediator of stress commonly used as a model of depressive-like behavior^41^. First, CORT induced a similar upregulation of CE and TG in both hippocampal poles (Fig. 3). Interestingly, accumulation of CE is increasingly seen as a hallmark of neurodegeneration. Although, the mechanism for neutral lipid accumulation remains elusive, lipid droplet formation reportedly mitigates lipotoxicity, oxidative damage and prevents neuronal loss^8,42–44^. Merril et al.^40^ report a partial enrichment of CE in neuron cell bodies relatively to brain parenchyma, whereas other studies implicate glial accumulation of lipid droplets in a non-autonomous fashion as a trigger for neuronal death^45^. In fact, both astrocytes and microglia are also subject to GC signaling and may hence contribute to the observed changes induced by CORT^46,47^. Simultaneously, we observed distinct regional responses to CORT, including an increase in longer Cer C22–26 species as well as dhSM, specifically in the ventral hippocampus, whereas the dorsal hippocampus showed increased HexCer levels (Fig. 4a). These results indicate contrasting dorsal-ventral sphingolipid catabolism rather than de novo synthesis under elevated CORT levels, with a putative increase in sphingomyelinase and hydroxilase activity, promoting recycling of long Cer species in the ventral hippocampus and inhibited glucocerebrosidase and galactosidase activity in the dorsal hippocampus. In fact, previous studies implicate the sphingomyelinase-ceramide pathway in stress-induced models of depression^26^. Acid and neutral sphingomyelinases (ASM and NSM, respectively) are inhibited and activated in the dorsal and ventral hippocampus, respectively, in hippocampal-dependent learning, with decreased Cer 24:0 and 24:1 in ventral hippocampus during behavior extinction^20^. While CORT treatment decreased extinction, we detected an increase in Cer 24:1 upon CORT treatment as well as significantly impaired biochemical coherence of this Cer species when analyzing the dorsal-ventral ratio before and post treatment (Fig. 4b). Whereas increased ASM activity correlates with neurodegeneration and depressive-like behavior, GC reportedly regulate sphingolipid enzyme transcription and induce Cer accumulation, which is linked to altered cellular signaling via activation of protein phosphatase 2A and dephosphorylation of pro-survival Akt^48,49^. Considering GC promote gene transcription in a cell-specific manner through binding to GC response elements, a thorough search of transcription regulatory elements will be required to understand how GC modulate lipid metabolism, either directly through enzyme transcription or post-translational modifications^50^. This metabolic pathway is hence a suggested player in cognition and mood regulation, with both pathological and therapeutical relevance, particularly considering Cer levels can be targeted by the direct action of antidepressants^51^.

We also found that CORT treatment differently modulated glycerophospholipids, namely upregulating PA species in the ventral hippocampus. Our analysis revealed a partially overlapping effect of CORT, with PA 32:1 and PA 34:2 equally increased in both hippocampal poles. Not only changes in ventral hippocampus were of higher magnitude, PA 34:1 was also increased (Fig. 4a). DG species, a product of PA dephosphorylation, were also increased to a greater extent in the ventral hippocampus (Fig. 4), whereas levels of long, unsaturated PC species were specifically affected (Fig. 4a). Two possible mechanisms may account for these observations. First, PC cleavage by phospholipase D (PLD), namely isoenzymes 1 and 2, may account for increased levels in PA^52^. Previous studies linked PLD2 activity to modulation of PA C32–34, suggesting more prominent PLD2 recruitment in the ventral hippocampus upon CORT treatment^53,54^. However, increased levels of DG also suggest increased PA dephosphorylation, either as a compensatory buffering mechanism to normalize PA levels, or de novo synthesis^55^. Beyond the important role as fundamental precursors for phospholipid synthesis, DG and PA also act as second messengers and modulate synaptic plasticity, possibly contributing to dorsal-ventral functional dichotomy^55,56^. Finally, we detected a common increase in shorter and less-unsaturated PI species (34:0, 34:1, 36:1, and 36:2) in both regions. Of note, PI species not containing arachidonic or stearic acid (20:4 and 18:0, respectively) not only are less abundant but also not as readily convertible to polyphosphorylation, possibly inhibiting cellular signaling (*e.g.*, via Akt)^57,58^. A possible explanation for such observation is distinct acyl chain incorporation in PI synthesis by enzyme modulation (DG kinases or CDP-diacylglycerol synthases). However, considering the low abundance of these species, acyl chain enrichment may instead depend on subcellular lipid remodeling, namely via inhibition of acyltransferases, such as LYCAT and LPIAT1, responsible for incorporating stearic and arachidonic acid, respectively^59,60^. Considering that manipulation of PI acyl chain disrupts brain morphology and function, future studies should address the abundance of free fatty acids to clarify substrate availability for lipid mediators^61,62^.

In summary, our study reveals a distinct lipid composition along the longitudinal axis of the hippocampus. We validate the ventral hippocampus as more sensitive to lipid modulation by CORT, in line with previous reports of increased regional synaptic plasticity and susceptibility to other stressors, such as kainate-induced seizures^14,63,64^. Thus, the distribution of lipids and enzymes across brain regions may determine regional susceptibility to distinct neurological disorders. This view privileges the prioritization of designed therapeutical approaches for different conditions using enzyme inhibition, replacement or silencing therapies or dietary manipulations^65^. A currently promising approach is the modulation of sphingolipids and glycerophospholipids in mood disorders, namely through pharmacological usage of functional inhibitors of acid sphingomyelinase and antidepressants, respectively^66,67^. Altogether, a thorough knowledge of hippocampal subregional identity will provide new tools to understand and manipulate each region’s function, both in health and disease.

## Supplementary information

Supplemental Figure 1

Supplemental Figure 2

Supplemental Figure 3

Supplemental Figure Legends and Supplementary Tables

## References

[1] Piomelli D, Astarita G, Rapaka R (2007). A neuroscientist’s guide to lipidomics. Nat. Rev. Neurosci..

[2] Wenk MR (2010). Lipidomics: new tools and applications. Cell.

[3] Miranda AM, Oliveira TG (2015). Lipids under stress-a lipidomic approach for the study of mood disorders. BioEssays.

[4] Brügger B (2014). Lipidomics: analysis of the lipid composition of cells and subcellular organelles by electrospray ionization mass spectrometry. Annu Rev. Biochem..

[5] Aureli M, Grassi S, Prioni S, Sonnino S, Prinetti A (2015). Lipid membrane domains in the brain. Biochim. Biophys. Acta.

[6] Cermenati G (2015). Lipids in the nervous system: From biochemistry and molecular biology to patho-physiology. Biochim. Biophys. Acta.

[7] Bozek K (2015). Organization and evolution of brain lipidome revealed by large-scale analysis of human, chimpanzee, macaque, and mouse tissues. Neuron.

[8] Chan RB (2012). Comparative lipidomic analysis of mouse and human brain with Alzheimer disease. J. Biol. Chem..

[9] Mapstone M (2014). Plasma phospholipids identify antecedent memory impairment in older adults. Nat. Med..

[10] Strange Ba, Witter MP, Lein ES, Moser EI (2014). Functional organization of the hippocampal longitudinal axis. Nat. Rev. Neurosci..

[11] Kheirbek MA (2013). Differential control of learning and anxiety along the dorsoventral axis of the dentate gyrus. Neuron.

[12] McHugh SB, Fillenz M, Lowry JP, Rawlins JNP, Bannerman DM (2011). Brain tissue oxygen amperometry in behaving rats demonstrates functional dissociation of dorsal and ventral hippocampus during spatial processing and anxiety. Eur. J. Neurosci..

[13] Bannerman DM (2014). Hippocampal synaptic plasticity, spatial memory and anxiety. Nat. Rev. Neurosci..

[14] Pinto V (2015). Differential impact of chronic stress along the hippocampal dorsal–ventral axis. Brain Struct. Funct..

[15] Maggio N, Segal M (2007). Unique regulation of long term potentiation in the rat ventral hippocampus. Hippocampus.

[16] Thompson CL (2008). Genomic anatomy of the hippocampus. Neuron.

[17] Shah S, Lubeck E, Zhou W, Cai L (2017). seqFISH accurately detects transcripts in single cells and reveals robust spatial organization in the hippocampus. Neuron.

[18] Bienkowski M. S., et al. Integration of gene expression and brain-wide connectivity reveals the multiscale organization of mouse hippocampal networks. *Nat. Neurosci.* **21**, 1628–1643 (2018). 10.1038/s41593-018-0241-y PMC6398347 30297807

[19] Müller CP (2014). Brain membrane lipids in major depression and anxiety disorders. Biochim. Biophys. Acta.

[20] Huston JP (2016). A sphingolipid mechanism for behavioral extinction. J. Neurochem..

[21] McEwen BS, Gianaros PJ (2011). Stress- and allostasis-induced brain plasticity. Annu. Rev. Med..

[22] Lucassen PJ (2014). Neuropathology of stress. Acta Neuropathol..

[23] Sousa Nuno, Almeida Osborne F.X. (2012). Disconnection and reconnection: the morphological basis of (mal)adaptation to stress. Trends in Neurosciences.

[24] Segal M, Richter-Levin G, Maggio N (2010). Stress-induced dynamic routing of hippocampal connectivity: a hypothesis. Hippocampus.

[25] Sousa N, Cerqueira JJ, Almeida OFX (2008). Corticosteroid receptors and neuroplasticity. Brain Res. Rev..

[26] Oliveira TG (2016). The impact of chronic stress on the rat brain lipidome. Mol. Psychiatry.

[27] Liebisch Gerhard, Vizcaíno Juan Antonio, Köfeler Harald, Trötzmüller Martin, Griffiths William J., Schmitz Gerd, Spener Friedrich, Wakelam Michael J. O. (2013). Shorthand notation for lipid structures derived from mass spectrometry. Journal of Lipid Research.

[28] Fahy E (2005). A comprehensive classification system for lipids. J. Lipid Res..

[29] Holthuis JCM, Menon AK (2014). Lipid landscapes and pipelines in membrane homeostasis. Nature.

[30] Sousa N, Lukoyanov N, Madeira M, Almeida O, Paula-Barbosa M (2000). Reorganization of the morphology of hippocampal neurites and synapses after stress-induced damage correlates with behavioral improvement. Neuroscience.

[31] Cerqueira JJ, Mailliet F, Almeida OFX, Jay TM, Sousa N (2007). The prefrontal cortex as a key target of the maladaptive response to stress. J. Neurosci..

[32] Wegner MS, Schiffmann S, Parnham MJ, Geisslinger G, Grösch S (2016). The enigma of ceramide synthase regulation in mammalian cells. Prog. Lipid Res..

[33] Lee AR, Kim JH, Cho E, Kim M, Park M (2017). Dorsal and ventral hippocampus differentiate in functional pathways and differentially associate with neurological disease-related genes during postnatal development. Front. Mol. Neurosci..

[34] Ebel Philipp, vom Dorp Katharina, Petrasch-Parwez Elisabeth, Zlomuzica Armin, Kinugawa Kiyoka, Mariani Jean, Minich David, Ginkel Christina, Welcker Jochen, Degen Joachim, Eckhardt Matthias, Dere Ekrem, Dörmann Peter, Willecke Klaus (2013). Inactivation of Ceramide Synthase 6 in Mice Results in an Altered Sphingolipid Metabolism and Behavioral Abnormalities. Journal of Biological Chemistry.

[35] Ginkel C (2012). Ablation of neuronal ceramide synthase 1 in mice decreases ganglioside levels and expression of myelin-associated glycoprotein in oligodendrocytes. J. Biol. Chem..

[36] Hannun YA, Obeid LM (2011). Many ceramides. J. Biol. Chem..

[37] Hanrieder J, Phan NTN, Kurczy ME, Ewing AG (2013). Imaging mass spectrometry in neuroscience. ACS Chem. Neurosci..

[38] Nielsen MMB (2016). Mass spectrometry imaging of biomarker lipids for phagocytosis and signalling during focal cerebral ischaemia. Sci. Rep..

[39] Zhang Y (2014). An RNA-sequencing transcriptome and splicing database of glia, neurons, and vascular cells of the cerebral cortex. J. Neurosci..

[40] Merrill CB (2017). Patch clamp-assisted single neuron lipidomics. Sci. Rep..

[41] Sterner EY, Kalynchuk LE (2010). Behavioral and neurobiological consequences of prolonged glucocorticoid exposure in rats: relevance to depression. Prog. Neuro-Psychopharmacol. Biol. Psychiatry.

[42] Rodriguez-Navarro JA (2012). Inhibitory effect of dietary lipids on chaperone-mediated autophagy. Proc. Natl. Acad. Sci. USA.

[43] Miranda AM (2018). Neuronal lysosomal dysfunction releases exosomes harboring APP C-terminal fragments and unique lipid signatures. Nat. Commun..

[44] Welte MA (2015). Expanding roles for lipid droplets. Curr. Biol..

[45] Liu L (2015). Glial lipid droplets and ROS induced by mitochondrial defects promote neurodegeneration. Cell.

[46] Bridges N, Slais K, Syková E (2008). The effects of chronic corticosterone on hippocampal astrocyte numbers: a comparison of male and female Wistar rats. Acta Neurobiol. Exp. (Wars.).

[47] Carter BS, Hamilton DE, Thompson RC (2013). Acute and chronic glucocorticoid treatments regulate astrocyte-enriched mRNAs in multiple brain regions in vivo. Front. Neurosci..

[48] Holland WL (2007). Inhibition of ceramide synthesis ameliorates glucocorticoid-, saturated-fat-, and obesity-induced insulin resistance. Cell Metab..

[49] Gulbins E (2013). Acid sphingomyelinase-ceramide system mediates effects of antidepressant drugs. Nat. Med..

[50] Datson NA, Morsink MC, Meijer OC, de Kloet ER (2008). Central corticosteroid actions: search for gene targets. Eur. J. Pharm..

[51] Kornhuber J, Müller CP, Becker KA, Reichel M, Gulbins E (2014). The ceramide system as a novel antidepressant target. Trends Pharm. Sci..

[52] Oliveira TG, Di Paolo G (2010). Phospholipase D in brain function and Alzheimer’s disease. Biochim. Biophys. Acta.

[53] Oliveira TG (2010). Phospholipase D2 ablation ameliorates Alzheimer’s disease-linked synaptic dysfunction and cognitive deficits. J. Neurosci..

[54] Vermeren MM (2016). The phospholipase D2 knock out mouse has ectopic purkinje cells and suffers from early adult-onset anosmia. PLoS ONE.

[55] Carrasco S, Mérida I (2007). Diacylglycerol, when simplicity becomes complex. Trends Biochem. Sci..

[56] Lee D, Kim E, Tanaka-Yamamoto K (2016). Diacylglycerol kinases in the coordination of synaptic plasticity. Front.Cell Dev. Biol..

[57] Epand RM (2017). Features of the phosphatidylinositol cycle and its role in signal transduction. J. Membr. Biol..

[58] Traynor-Kaplan A (2017). Fatty-acyl chain profiles of cellular phosphoinositides. Biochim. Biophys. Acta.

[59] Lee H (2012). LPIAT1 regulates arachidonic acid content in phosphatidylinositol and is required for cortical lamination in mice. Mol. Biol. Cell.

[60] Imae R (2012). LYCAT, a homologue of *C. elegans acl-8*, *acl-9*, and *acl-10*, determines the fatty acid composition of phosphatidylinositol in mice. J. Lipid Res..

[61] Robichaud PP, Surette ME (2015). Polyunsaturated fatty acid-phospholipid remodeling and inflammation. Curr. Opin. Endocrinol. Diabetes Obes..

[62] D’Souza K, Epand RM (2014). Enrichment of phosphatidylinositols with specific acyl chains. Biochim. Biophys. Acta.

[63] Maggio N, Segal M (2007). Striking variations in corticosteroid modulation of long-term potentiation along the septotemporal axis of the hippocampus. J. Neurosci..

[64] Lerner R (2018). Simultaneous lipidomic and transcriptomic profiling in mouse brain punches of acute epileptic seizure model compared to controls. J. Lipid Res..

[65] Schneider M (2017). Lipids in psychiatric disorders and preventive medicine. Neurosci. Biobehav. Rev..

[66] Müller CP (2017). Paradoxical antidepressant effects of alcohol are related to acid sphingomyelinase and its control of sphingolipid homeostasis. Acta Neuropathol..

[67] Aboukhatwa MA, Undieh AS (2010). Antidepressant stimulation of CDP-diacylglycerol synthesis does not require monoamine reuptake inhibition. BMC Neurosci..

