# Supplementary figures and images for: Differential lipid composition and regulation along the hippocampal longitudinal axis
Source: Transl Psychiatry. 2019 Apr 26;9:144. doi: 10.1038/s41398-019-0478-6 (PMC6486574; doi:10.1038/s41398-019-0478-6)

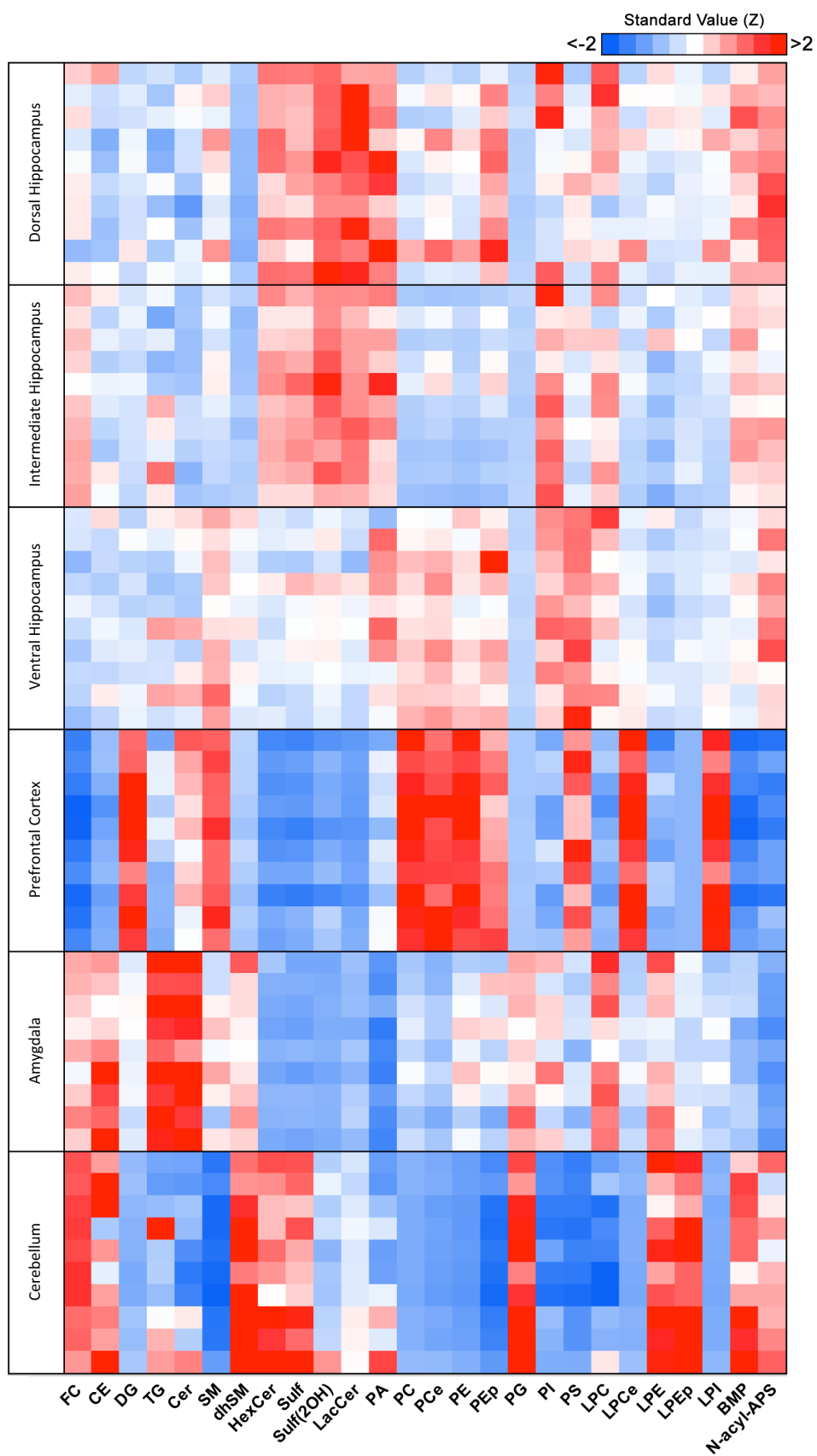

Supplementary Figure 1 – Miranda et al.

Supplement: Supplementary file 1 — Supplemental Figure 1 [file 41398_2019_478_MOESM1_ESM.pdf]

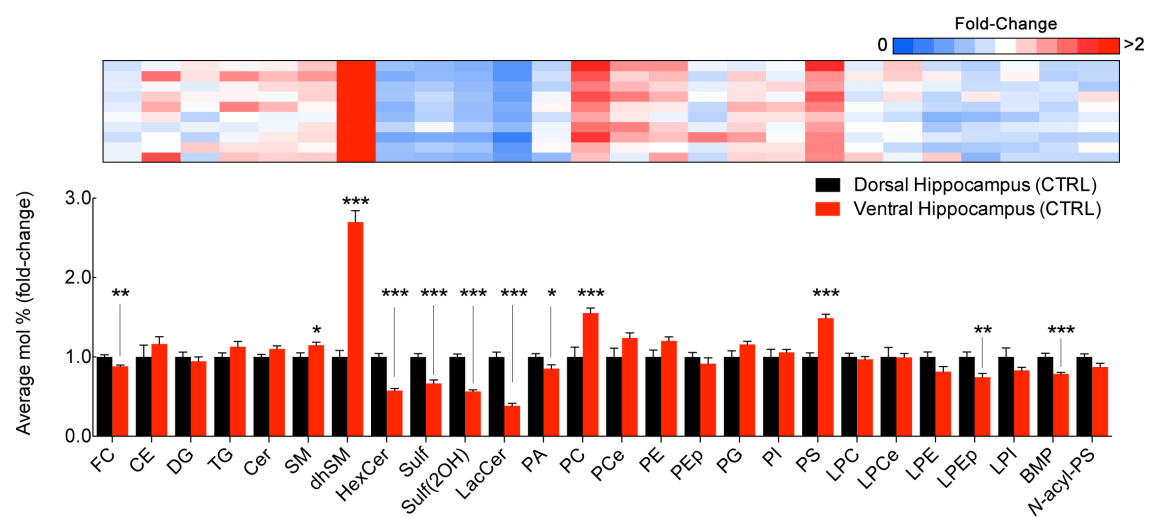

Supplementary Figure 2 – Miranda et al.

Supplement: Supplementary file 2 — Supplemental Figure 2 [file 41398_2019_478_MOESM2_ESM.pdf]

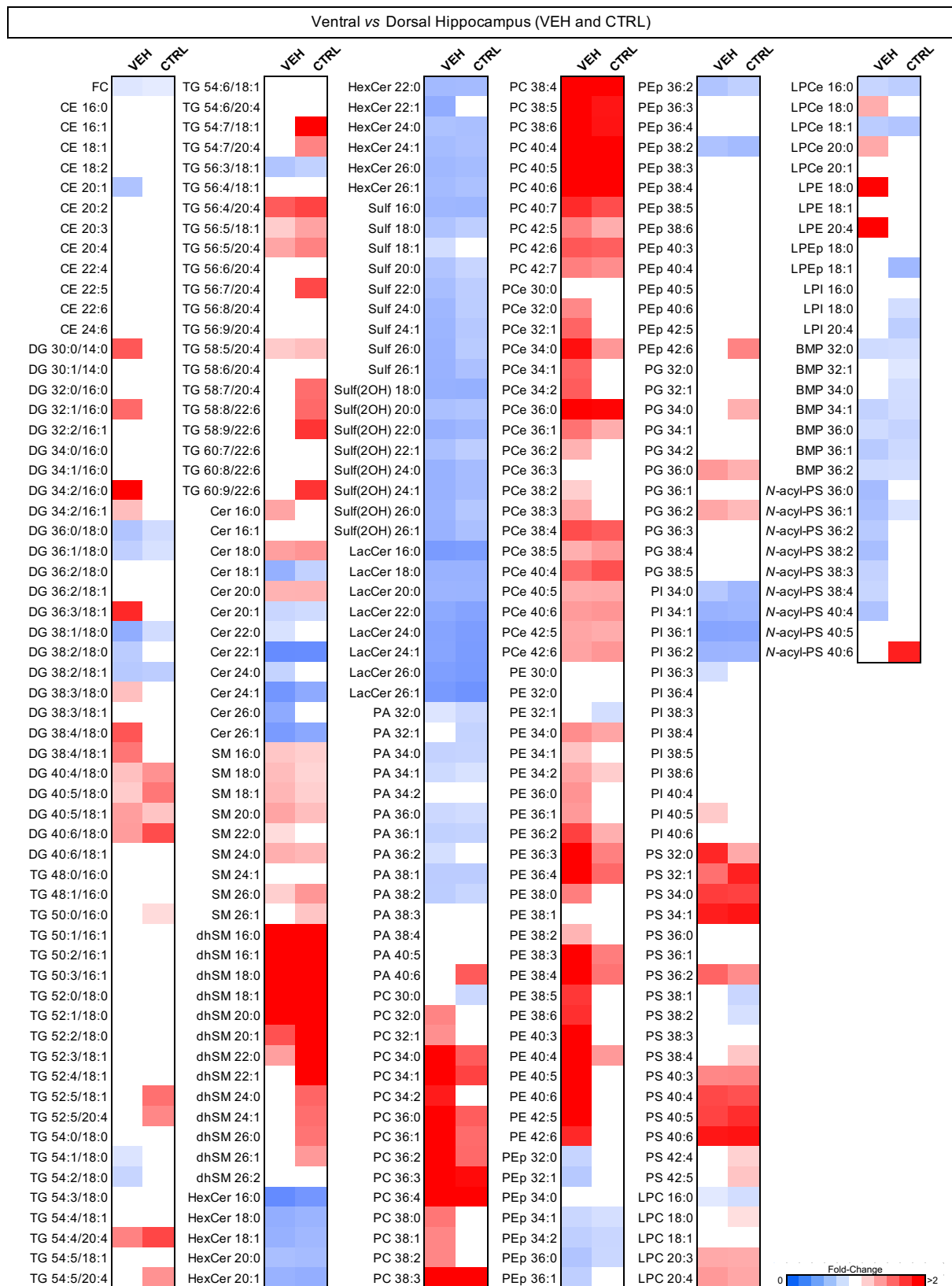

Supplementary Figure 3 – Miranda *et al.*

Supplement: Supplementary file 3 — Supplemental Figure 3 [file 41398_2019_478_MOESM3_ESM.pdf]
