# Supplementary material for: Differential lipid composition and regulation along the hippocampal longitudinal axis
Source: Transl Psychiatry. 2019 Apr 26;9:144. doi: 10.1038/s41398-019-0478-6 (PMC6486574; doi:10.1038/s41398-019-0478-6)
Supplement: Supplementary file 4 — Supplemental Figure Legends and Supplementary Tables [file 41398_2019_478_MOESM4_ESM.docx]

**Supplementary Fig. 1** Lipidomic analysis of brain regions from control adult rats (not injected; CTRL). Adult rats were handled daily injections and homogenates of the following brain regions were analyzed by LC-MS (see Figure 1). For lipid nomenclature, see Methods section. Heatmap generated by calculating the standard value of each lipid class (column) using as reference the pooled average relative mol% of all brain regions. Z values [(mol% of lipid class of each animal – average mol% of lipid class in all brain regions of pool of animals)/standard deviation of average mol% lipid class of all brain regions of pool of animals] represented in gradient color; blue indicates negative Z value; red indicates positive value (lower and higher than reference average, respectively). Each row indicates an individual animal, per brain region (N=10 animals).

**Supplementary Fig 2.** Distinct lipid composition of dorsal and ventral hippocampus in control adult rats (CTRL). LC-MS analysis of dorsal and ventral hippocampus macrodissected from adult rats (not injected; CTRL). For lipid nomenclature, see Methods section. Bar graphs indicate fold-change of average relative mol% of all lipids measured, normalized to dorsal hippocampus (mean ± SEM, *N*= 10 per group). Upper panel, Upper panel, heatmap indicates individual plot of normalized average mol% of each lipid class in the ventral hippocampus. Values represented in gradient color, blue indicates below 1 and red indicates above 1 fold-change, respectively, normalized to dorsal hippocampus. Descriptive statistics listed in Supplementary Table 2. **p*< 0.05, ***p*< 0.01 and ***p<0.001 in two-tailed Student’s *t*-test.

**Supplementary Fig 3.** Comparative lipidome profile of dorsal and ventral hippocampus in adult rats injected with vehicle (VEH) and control adult rats (CTRL). The heatmap represents all lipid species detected by LC-MS in macro-dissected tissue. Lipids were classified per total acyl carbons and degree of unsaturation, respectively. Values represented as average fold-change of individual species in ventral hippocampus, normalized to dorsal hippocampus (only statistically significant values shown (p<0.05), N=9 for dorsal hippocampus in vehicle injected rats; N=10 for remaining groups). Results were compared using two-tailed Student’s *t*-test.

**Supplementary Table 1.** Descriptive statistics of lipidomic analysis of dorsal and ventral hippocampus in adult rats injected with vehicle (VEH) (see Fig. 2a).

|  | **Average mol % ± SEM** | | | | | |  |
| --- | --- | --- | --- | --- | --- | --- | --- |
|  | VEH Dorsal (N=9) | | | VEH Ventral (N=10) | | | ***p* value** |
| **FC** | 66,353 | ± | 1,643 | 55,919 | ± | 1,409 | 0,0001507 |
| **CE** | 0,401 | ± | 0,043 | 0,465 | ± | 0,035 | 0,254838 |
| **DG** | 0,317 | ± | 0,023 | 0,368 | ± | 0,014 | 0,0699443 |
| **TG** | 0,055 | ± | 0,003 | 0,057 | ± | 0,003 | 0,526496 |
| **Cer** | 0,176 | ± | 0,005 | 0,193 | ± | 0,009 | 0,130663 |
| **SM** | 3,235 | ± | 0,150 | 4,050 | ± | 0,118 | 0,0004716 |
| **dhSM** | 0,344 | ± | 0,027 | 0,711 | ± | 0,035 | 2,523E-07 |
| **HexCer** | 0,804 | ± | 0,051 | 0,450 | ± | 0,023 | 5,449E-06 |
| **Sulf** | 1,044 | ± | 0,061 | 0,565 | ± | 0,036 | 2,56E-06 |
| **Sulf(2OH)** | 0,695 | ± | 0,023 | 0,352 | ± | 0,018 | 1,148E-09 |
| **LacCer** | 0,119 | ± | 0,007 | 0,051 | ± | 0,006 | 6,073E-07 |
| **PA** | 0,431 | ± | 0,031 | 0,348 | ± | 0,017 | 0,0278639 |
| **PC** | 7,369 | ± | 0,701 | 15,178 | ± | 0,973 | 6,836E-06 |
| **PCe** | 0,490 | ± | 0,036 | 0,768 | ± | 0,045 | 0,0001685 |
| **PE** | 1,296 | ± | 0,112 | 2,125 | ± | 0,157 | 0,0005901 |
| **PEp** | 6,752 | ± | 0,710 | 5,947 | ± | 0,206 | 0,269989 |
| **PG** | 0,113 | ± | 0,008 | 0,129 | ± | 0,010 | 0,245009 |
| **PI** | 4,390 | ± | 0,164 | 4,166 | ± | 0,153 | 0,331351 |
| **PS** | 5,284 | ± | 0,526 | 7,804 | ± | 0,197 | 0,0002169 |
| **LPC** | 0,128 | ± | 0,002 | 0,127 | ± | 0,006 | 0,892517 |
| **LPCe** | 0,018 | ± | 0,001 | 0,022 | ± | 0,001 | 0,0095462 |
| **LPE** | 0,034 | ± | 0,003 | 0,057 | ± | 0,004 | 0,00015 |
| **LPEp** | 0,041 | ± | 0,004 | 0,038 | ± | 0,002 | 0,544033 |
| **LPI** | 0,072 | ± | 0,006 | 0,084 | ± | 0,005 | 0,169681 |
| **BMP** | 0,011 | ± | 0,000 | 0,009 | ± | 0,000 | 6,324E-05 |
| **APS** | 0,028 | ± | 0,001 | 0,017 | ± | 0,001 | 1,157E-05 |

**Supplementary Table 2.** Descriptive statistics of lipidomic analysis of dorsal and ventral hippocampus in adult rats (not injected; CTRL) (see Supplementary Fig. 2).

|  | **Average mol % ± SEM** | | | | | |  |
| --- | --- | --- | --- | --- | --- | --- | --- |
|  | CTRL Dorsal (N=10) | | | CTRL Ventral (N=10) | | | ***p* value** |
| **FC** | 63,671 | ± | 1,748 | 56,054 | ± | 1,153 | 0,0018837 |
| **CE** | 0,434 | ± | 0,065 | 0,505 | ± | 0,039 | 0,358368 |
| **DG** | 0,354 | ± | 0,021 | 0,335 | ± | 0,020 | 0,518469 |
| **TG** | 0,055 | ± | 0,003 | 0,062 | ± | 0,004 | 0,133091 |
| **Cer** | 0,179 | ± | 0,006 | 0,197 | ± | 0,007 | 0,0630298 |
| **SM** | 3,756 | ± | 0,192 | 4,307 | ± | 0,150 | 0,0364722 |
| **dhSM** | 0,315 | ± | 0,026 | 0,849 | ± | 0,045 | 6,288E-09 |
| **HexCer** | 0,983 | ± | 0,044 | 0,567 | ± | 0,027 | 2,143E-07 |
| **Sulf** | 1,041 | ± | 0,044 | 0,694 | ± | 0,046 | 3,444E-05 |
| **Sulf(2OH)** | 0,699 | ± | 0,026 | 0,395 | ± | 0,015 | 7,086E-09 |
| **LacCer** | 0,161 | ± | 0,010 | 0,062 | ± | 0,005 | 4,952E-08 |
| **PA** | 0,393 | ± | 0,017 | 0,335 | ± | 0,019 | 0,0347713 |
| **PC** | 9,729 | ± | 1,206 | 15,109 | ± | 0,632 | 0,0009374 |
| **PCe** | 0,635 | ± | 0,071 | 0,787 | ± | 0,041 | 0,0825695 |
| **PE** | 1,635 | ± | 0,144 | 1,965 | ± | 0,083 | 0,0626995 |
| **PEp** | 7,096 | ± | 0,403 | 6,496 | ± | 0,516 | 0,371483 |
| **PG** | 0,109 | ± | 0,009 | 0,126 | ± | 0,005 | 0,0970039 |
| **PI** | 3,785 | ± | 0,364 | 4,009 | ± | 0,133 | 0,570729 |
| **PS** | 4,572 | ± | 0,231 | 6,799 | ± | 0,231 | 2,2E-06 |
| **LPC** | 0,129 | ± | 0,006 | 0,125 | ± | 0,005 | 0,614326 |
| **LPCe** | 0,022 | ± | 0,003 | 0,021 | ± | 0,001 | 0,969814 |
| **LPE** | 0,056 | ± | 0,004 | 0,046 | ± | 0,004 | 0,0561738 |
| **LPEp** | 0,053 | ± | 0,003 | 0,039 | ± | 0,003 | 0,0045292 |
| **LPI** | 0,097 | ± | 0,011 | 0,081 | ± | 0,004 | 0,17867 |
| **BMP** | 0,012 | ± | 0,001 | 0,009 | ± | 0,000 | 0,0004428 |
| **APS** | 0,029 | ± | 0,001 | 0,026 | ± | 0,001 | 0,0555445 |

**Supplementary Table 3.** Descriptive statistics of diacylglycerol/glycerophospholipid and sphingolipid acyl chain analysis from dorsal and ventral hippocampus in adult rats injected with vehicle (VEH) (see Fig. 2b).

|  |  |  | **Average mol % ± SEM** | | | | | |  |
| --- | --- | --- | --- | --- | --- | --- | --- | --- | --- |
|  |  |  | VEH Dorsal (N=9) | | | VEH Ventral (N=10) | | | ***p* value** |
| DG/Glycerophospholipids | FA Length | **<32** | 10,300 | ± | 0,555 | 10,340 | ± | 0,185 | 0,943858 |
|  |  | **34** | 14,400 | ± | 0,314 | 16,040 | ± | 0,126 | 0,0001006 |
|  |  | **36** | 26,144 | ± | 0,368 | 22,680 | ± | 0,212 | 1,964E-07 |
|  |  | **38** | 29,122 | ± | 0,854 | 26,760 | ± | 0,231 | 0,0122708 |
|  |  | **>40** | 20,033 | ± | 0,742 | 24,170 | ± | 0,284 | 4,581E-05 |
|  | Saturation | **0** | 13,133 | ± | 0,582 | 13,560 | ± | 0,208 | 0,481353 |
|  |  | **1** | 25,056 | ± | 0,516 | 23,430 | ± | 0,293 | 0,0119897 |
|  |  | **2** | 6,978 | ± | 0,280 | 5,220 | ± | 0,105 | 1,121E-05 |
|  |  | **3** | 3,867 | ± | 0,120 | 3,600 | ± | 0,033 | 0,0386491 |
|  |  | **4** | 25,678 | ± | 0,839 | 24,890 | ± | 0,224 | 0,354454 |
|  |  | **5** | 7,300 | ± | 0,169 | 7,590 | ± | 0,064 | 0,113276 |
|  |  | **>6** | 17,933 | ± | 0,517 | 21,700 | ± | 0,233 | 2,642E-06 |
| Sphingolipids | FA Length | **16** | 5,833 | ± | 0,155 | 6,960 | ± | 0,119 | 2,074E-05 |
|  |  | **18** | 34,756 | ± | 1,201 | 44,620 | ± | 0,992 | 6,846E-06 |
|  |  | **20** | 8,633 | ± | 0,254 | 11,670 | ± | 0,211 | 4,647E-08 |
|  |  | **22** | 7,900 | ± | 0,250 | 6,190 | ± | 0,143 | 1,202E-05 |
|  |  | **24** | 40,011 | ± | 1,160 | 28,600 | ± | 0,936 | 5,861E-07 |
|  |  | **26** | 2,889 | ± | 0,081 | 1,970 | ± | 0,092 | 9,744E-07 |
|  | Sat. | **0** | 73,244 | ± | 0,725 | 79,170 | ± | 0,527 | 3,671E-06 |
|  |  | **1** | 26,756 | ± | 0,725 | 20,830 | ± | 0,527 | 3,671E-06 |

**Supplementary Table 4.** Descriptive statistics of lipidomic analysis of dorsal and ventral hippocampus in adult rats submitted to a 4-week protocol of daily injections of corticoterone (CORT) compared to age-matched control injected with vehicle (VEH) (see Fig. 3).

|  | **Average mol % ± SEM** | | | | | |  |  | **Average mol % ± SEM** | | | | | |  |
| --- | --- | --- | --- | --- | --- | --- | --- | --- | --- | --- | --- | --- | --- | --- | --- |
|  | VEH Dorsal (N=9) | | | CORT Dorsal (N=10) | | | ***p* value** |  | VEH Ventral (N=10) | | | CORT Ventral (N=10) | | | ***p* value** |
| **FC** | 66,353 | ± | 1,643 | 67,230 | ± | 1,373 | 0,685404 |  | 55,919 | ± | 1,409 | 55,286 | ± | 1,725 | 0,779709 |
| **CE** | 0,401 | ± | 0,043 | 0,733 | ± | 0,088 | 0,0044823 |  | 0,465 | ± | 0,035 | 0,785 | ± | 0,084 | 0,0024302 |
| **DG** | 0,317 | ± | 0,023 | 0,302 | ± | 0,016 | 0,597042 |  | 0,368 | ± | 0,014 | 0,421 | ± | 0,031 | 0,138803 |
| **TG** | 0,055 | ± | 0,003 | 0,062 | ± | 0,004 | 0,155667 |  | 0,057 | ± | 0,003 | 0,073 | ± | 0,005 | 0,0135612 |
| **Cer** | 0,176 | ± | 0,005 | 0,177 | ± | 0,004 | 0,886283 |  | 0,193 | ± | 0,009 | 0,204 | ± | 0,008 | 0,380811 |
| **SM** | 3,235 | ± | 0,150 | 3,210 | ± | 0,127 | 0,900362 |  | 4,050 | ± | 0,118 | 4,138 | ± | 0,244 | 0,748007 |
| **dhSM** | 0,344 | ± | 0,027 | 0,379 | ± | 0,032 | 0,415053 |  | 0,711 | ± | 0,035 | 0,811 | ± | 0,028 | 0,0389376 |
| **HexCer** | 0,804 | ± | 0,051 | 0,937 | ± | 0,049 | 0,078002 |  | 0,450 | ± | 0,023 | 0,457 | ± | 0,035 | 0,879128 |
| **Sulf** | 1,044 | ± | 0,061 | 1,094 | ± | 0,046 | 0,514374 |  | 0,565 | ± | 0,036 | 0,590 | ± | 0,062 | 0,740295 |
| **Sulf(2OH)** | 0,695 | ± | 0,023 | 0,698 | ± | 0,025 | 0,945748 |  | 0,352 | ± | 0,018 | 0,326 | ± | 0,022 | 0,36901 |
| **LacCer** | 0,119 | ± | 0,007 | 0,138 | ± | 0,007 | 0,053735 |  | 0,051 | ± | 0,006 | 0,048 | ± | 0,004 | 0,67821 |
| **PA** | 0,431 | ± | 0,031 | 0,406 | ± | 0,020 | 0,494921 |  | 0,348 | ± | 0,017 | 0,377 | ± | 0,019 | 0,268389 |
| **PC** | 7,369 | ± | 0,701 | 7,373 | ± | 0,968 | 0,997266 |  | 15,178 | ± | 0,973 | 14,153 | ± | 1,057 | 0,484607 |
| **PCe** | 0,490 | ± | 0,036 | 0,462 | ± | 0,058 | 0,702554 |  | 0,768 | ± | 0,045 | 0,675 | ± | 0,056 | 0,211589 |
| **PE** | 1,296 | ± | 0,112 | 1,239 | ± | 0,083 | 0,683753 |  | 2,125 | ± | 0,157 | 1,886 | ± | 0,198 | 0,356705 |
| **PEp** | 6,752 | ± | 0,710 | 5,747 | ± | 0,394 | 0,220438 |  | 5,947 | ± | 0,206 | 5,802 | ± | 0,320 | 0,707067 |
| **PG** | 0,113 | ± | 0,008 | 0,117 | ± | 0,010 | 0,746664 |  | 0,129 | ± | 0,010 | 0,112 | ± | 0,009 | 0,228573 |
| **PI** | 4,390 | ± | 0,164 | 4,440 | ± | 0,378 | 0,909443 |  | 4,166 | ± | 0,153 | 4,412 | ± | 0,209 | 0,355043 |
| **PS** | 5,284 | ± | 0,526 | 4,908 | ± | 0,261 | 0,517486 |  | 7,804 | ± | 0,197 | 9,058 | ± | 0,232 | 0,000642 |
| **LPC** | 0,128 | ± | 0,002 | 0,116 | ± | 0,003 | 0,0081532 |  | 0,127 | ± | 0,006 | 0,134 | ± | 0,004 | 0,293243 |
| **LPCe** | 0,018 | ± | 0,001 | 0,017 | ± | 0,001 | 0,773878 |  | 0,022 | ± | 0,001 | 0,024 | ± | 0,002 | 0,668467 |
| **LPE** | 0,034 | ± | 0,003 | 0,043 | ± | 0,004 | 0,0862452 |  | 0,057 | ± | 0,004 | 0,062 | ± | 0,005 | 0,503134 |
| **LPEp** | 0,041 | ± | 0,004 | 0,053 | ± | 0,006 | 0,123359 |  | 0,038 | ± | 0,002 | 0,049 | ± | 0,007 | 0,14855 |
| **LPI** | 0,072 | ± | 0,006 | 0,075 | ± | 0,008 | 0,822832 |  | 0,084 | ± | 0,005 | 0,091 | ± | 0,010 | 0,513212 |
| **BMP** | 0,011 | ± | 0,000 | 0,009 | ± | 0,000 | 1,304E-05 |  | 0,009 | ± | 0,000 | 0,007 | ± | 0,000 | 0,0118198 |
| **APS** | 0,028 | ± | 0,001 | 0,035 | ± | 0,002 | 0,0034333 |  | 0,017 | ± | 0,001 | 0,021 | ± | 0,002 | 0,0800939 |

**Supplementary Table 5.** Descriptive statistics of diacylglycerol/glycerophospholipid and sphingolipid acyl chain analysis from dorsal and ventral hippocampus in adult rats submitted to a 4-week protocol of daily injections of corticoterone (CORT) compared to age-matched control injected with vehicle (VEH) (see Fig. 3).

|  |  |  | **Average mol % ± SEM** | | | | | |  |  | **Average mol % ± SEM** | | | | | |  |
| --- | --- | --- | --- | --- | --- | --- | --- | --- | --- | --- | --- | --- | --- | --- | --- | --- | --- |
|  |  |  | VEH Dorsal (N=9) | | | CORT Dorsal (N=10) | | | ***p* value** |  | VEH Ventral (N=10) | | | CORT Ventral (N=10) | | | ***p* value** |
| DG/Glycerophospholipids | FA Length | **<32** | 10,300 | ± | 0,555 | 10,800 | ± | 0,594 | 0,549115 |  | 10,340 | ± | 0,185 | 10,220 | ± | 0,234 | 0,692062 |
|  |  | **34** | 14,400 | ± | 0,314 | 14,970 | ± | 0,461 | 0,332211 |  | 16,040 | ± | 0,126 | 15,820 | ± | 0,289 | 0,494077 |
|  |  | **36** | 26,144 | ± | 0,368 | 26,490 | ± | 0,199 | 0,406835 |  | 22,680 | ± | 0,212 | 21,610 | ± | 0,578 | 0,099255 |
|  |  | **38** | 29,122 | ± | 0,854 | 28,590 | ± | 1,097 | 0,711093 |  | 26,760 | ± | 0,231 | 25,670 | ± | 0,343 | 0,0167662 |
|  |  | **>40** | 20,033 | ± | 0,742 | 19,150 | ± | 0,359 | 0,283493 |  | 24,170 | ± | 0,284 | 26,680 | ± | 0,646 | 0,0022491 |
|  | Saturation | **0** | 13,133 | ± | 0,582 | 13,470 | ± | 0,615 | 0,697615 |  | 13,560 | ± | 0,208 | 13,260 | ± | 0,237 | 0,354509 |
|  |  | **1** | 25,056 | ± | 0,516 | 25,690 | ± | 0,508 | 0,39418 |  | 23,430 | ± | 0,293 | 23,060 | ± | 0,686 | 0,625838 |
|  |  | **2** | 6,978 | ± | 0,280 | 6,940 | ± | 0,203 | 0,912972 |  | 5,220 | ± | 0,105 | 5,310 | ± | 0,174 | 0,66348 |
|  |  | **3** | 3,867 | ± | 0,120 | 3,980 | ± | 0,128 | 0,529964 |  | 3,600 | ± | 0,033 | 3,570 | ± | 0,060 | 0,666147 |
|  |  | **4** | 25,678 | ± | 0,839 | 25,500 | ± | 1,157 | 0,904375 |  | 24,890 | ± | 0,224 | 23,550 | ± | 0,413 | 0,0105307 |
|  |  | **5** | 7,300 | ± | 0,169 | 7,130 | ± | 0,082 | 0,36279 |  | 7,590 | ± | 0,064 | 7,530 | ± | 0,080 | 0,566469 |
|  |  | **>6** | 17,933 | ± | 0,517 | 17,280 | ± | 0,353 | 0,302783 |  | 21,700 | ± | 0,233 | 23,720 | ± | 0,610 | 0,0062354 |
| Sphingolipids | FA Length | **16** | 5,833 | ± | 0,155 | 6,070 | ± | 0,160 | 0,305532 |  | 6,960 | ± | 0,119 | 7,230 | ± | 0,409 | 0,533836 |
|  |  | **18** | 34,756 | ± | 1,201 | 33,590 | ± | 0,726 | 0,407073 |  | 44,620 | ± | 0,992 | 44,370 | ± | 1,620 | 0,896776 |
|  |  | **20** | 8,633 | ± | 0,254 | 9,250 | ± | 0,246 | 0,0997839 |  | 11,670 | ± | 0,211 | 12,080 | ± | 0,301 | 0,279284 |
|  |  | **22** | 7,900 | ± | 0,250 | 8,310 | ± | 0,149 | 0,166827 |  | 6,190 | ± | 0,143 | 6,640 | ± | 0,453 | 0,356537 |
|  |  | **24** | 40,011 | ± | 1,160 | 39,670 | ± | 0,684 | 0,798216 |  | 28,600 | ± | 0,936 | 27,650 | ± | 1,364 | 0,57293 |
|  |  | **26** | 2,889 | ± | 0,081 | 3,110 | ± | 0,115 | 0,141725 |  | 1,970 | ± | 0,092 | 2,010 | ± | 0,134 | 0,808089 |
|  | Sat. | **0** | 73,244 | ± | 0,725 | 73,580 | ± | 0,404 | 0,682911 |  | 79,170 | ± | 0,527 | 79,350 | ± | 0,875 | 0,862121 |
|  |  | **1** | 26,756 | ± | 0,725 | 26,420 | ± | 0,404 | 0,682912 |  | 20,830 | ± | 0,527 | 20,650 | ± | 0,875 | 0,86212 |
